# Aneuploidy is Linked to Neurological Phenotypes Through Oxidative Stress

**DOI:** 10.1007/s12031-024-02227-1

**Published:** 2024-05-02

**Authors:** Anowarul Islam, Zeeshan Shaukat, Rashid Hussain, Michael G. Ricos, Leanne M. Dibbens, Stephen L. Gregory

**Affiliations:** 1 Flinders Health and Medical Research Institute, College of Medicine and Public Health, Flinders University, Adelaide, 5042 Australia; 2 Epilepsy Research Group, Australian Centre for Precision Health, Clinical and Health Sciences, University of South Australia, Adelaide, South Australia 5000 Australia

**Keywords:** Aneuploidy, GABAergic neuron, Oxidative stress, Neurological phenotypes

## Abstract

Aneuploidy, having an aberrant genome, is gaining increasing attention in neurodegenerative diseases. It gives rise to proteotoxic stress as well as a stereotypical oxidative shift which makes these cells sensitive to internal and environmental stresses. A growing body of research from numerous laboratories suggests that many neurodegenerative disorders, especially Alzheimer’s disease and frontotemporal dementia, are characterised by neuronal aneuploidy and the ensuing apoptosis, which may contribute to neuronal loss. Using *Drosophila* as a model, we investigated the effect of induced aneuploidy in GABAergic neurons. We found an increased proportion of aneuploidy due to *Mad2* depletion in the third-instar larval brain and increased cell death. Depletion of *Mad2* in GABAergic neurons also gave a defective climbing and seizure phenotype. Feeding animals an antioxidant rescued the climbing and seizure phenotype. These findings suggest that increased aneuploidy leads to higher oxidative stress in GABAergic neurons which causes cell death, climbing defects, and seizure phenotype. Antioxidant feeding represents a potential therapy to reduce the aneuploidy-driven neurological phenotype.

**Supplementary Information:**

The online version contains supplementary material available at 10.1007/s12031-024-02227-1.

## Introduction

Aneuploidy is the state in which cells carry an abnormal DNA complement (Santaguida and Amon 2015). Generally, the distribution of aneuploid chromosomes varies only by one or a small number of chromosomes from the wild type. Numerous genetic and environmental causes can cause aneuploidy, which has long been recognised as a significant contributor to human diseases, particularly in the context of cancer. However, the potential role of aneuploidy in non-malignant disorders, such as neurological diseases, has gained increasing attention in recent years. Aneuploidy has been linked to an increased incidence of neurodegenerative diseases, and with our aging population, these diseases are becoming more common (Faggioli et al. 2011; Iourov et al. 2009). Aneuploidy is well tolerated in the young brain, but aneuploid neural cells are sensitive to age-related metabolic disorders and senescence that impact motor function and lifespan (Mirkovic et al. 2019) as well as mental health (Yurov et al. 2018). Aneuploidy has been observed in the neural tissues of people who have neurodegenerative diseases, raising the question of whether it contributes to the onset and development of these disorders.  

Owing to the in vivo inaccessibility of human brain tissue, several researchers used peripheral cells such as lymphocytes and fibroblasts to examine the association between genomic damage and neurodegenerative diseases such as Alzheimer’s disease (AD). Several studies have been published with a link between AD and enhanced peripheral aneuploidy (Buckton et al. 1982, 1983; Geller and Potter 1999; Matsuyama and Bohman 1988; Ward et al. 1979). Since then, a connection between aneuploidy and AD has been confirmed by various studies that show a role for aneuploidy, particularly hyperploidy, early in the family and sporadic AD (Boeras et al. 2008; Yang et al. 2003; Yurov et al. 2014). Alzheimer’s brain disease has been shown to have elevated levels of spontaneous aneuploidy (Arendt et al. 2017; Bajic et al. 2015; Yurov et al. 2019, 2014). Consistent with these findings, Alzheimer’s disease genes are involved in molecular mechanisms that cause changes in chromosome missegregation and aneuploidy (Granic et al. 2010). Even in the absence of disease, neurological aneuploidy is not rare. As many as a third of neuroblasts are aneuploid in developing mouse brains, a number which declines during development, but the remaining aneuploid neurons are incorporated into the circuitry (Kingsbury et al. 2005).

Despite the mounting evidence suggesting a connection between aneuploidy and neurological diseases, the specific mechanisms by which aneuploidy might lead to neurological phenotypes remain poorly understood. Therefore, understanding the role of aneuploidy in the progression of neurodegeneration is important for successful therapeutic interventions.

To investigate the mechanisms by which aneuploidy changes neurological phenotypes, we used *Drosophila* as a model to knock down *Mad2* specifically in GABAergic neurons. These cells are of particular interest as inhibitory neurons that are affected in epilepsy, Huntington’s, and other neurological diseases (Kleppner and Tobin 2001). *Mad2* (Mitotic Arrest Deficient 2) is an essential spindle assembly checkpoint (SAC) protein. The spindle assembly checkpoint, which guarantees normal chromosomal segregation during cell division, is a crucial regulatory checkpoint involved in preventing aneuploidy. Consequently, depletion of *Mad2* has been found to cause aneuploidy in a range of tissue types (Buffin et al. 2007; Lentini et al. 2012).

This study aims to address the gap in our understanding of how increased aneuploidy, following *Mad2* depletion, may contribute to the development of a neurological phenotype in a *Drosophila* model. We hypothesised that elevated aneuploidy induced by *Mad2* depletion could result in oxidative stress in neural tissues, ultimately leading to neurological dysfunction which might be rescued by feeding antioxidants. Antioxidants are endogenous or exogenous substances that either prevent the free radical formation or react with them to neutralise, potentially shielding the cell from oxidative damage. We can feed antioxidants that pass the blood–brain barrier (Moulton et al. 2021), and our previous published data revealed that antioxidant feeding can rescue cell death in other aneuploid cell types (Islam et al. 2023).

This study sheds light on the intricate molecular pathways that connect aneuploidy and neurobiology, with implications for future research in both model organisms and human neurodegenerative diseases.

## Materials and Methods

### Aneuploidy Analysis by Karyotyping

Third-instar larval brains were dissected in 1 × PBS, and then the brain was soaked in 0.5% sodium citrate for 5 min. After that, it was transferred to the 45% acetic acid for 2 min. Then each brain was moved to a drop of 60% acetic acid on a coverslip for 1 min. The labelled glass slide was gently placed on the cover slip, inverted, and then squashed very hard with a thumb under filter paper. Then the slide was quickly placed in liquid nitrogen for 10 min, and after that, slides were removed from liquid nitrogen, and the coverslip flicked off with a scalpel. Then slides were placed in 100% ethanol for 10 min after that tissue was washed with 1 × PBS and stained with Hoechst 33,342 solution (2 µg /ml) for 10 min. After staining, tissues were washed with 1 × PBS and mounted with 80% glycerol. Finally, slides were ready for taking pictures for karyotyping using the 20 × objective lens on an Olympus IX71 microscope.

### Cell Death Analysis by Hoechst Stain

The slides for cell death analysis were prepared as described above for aneuploidy analysis. The pictures were taken under a 20 × objective lens on an Olympus IX71 microscope. The bright, pyknotic, and round-shaped nuclei were considered to be undergoing cell death in our analysis, and we manually counted the numbers blinded for each genotype.

### *Drosophila* Stocks

*Drosophila* were cultured in 12-h light/dark cycles at 25 °C on a fortified medium (1% agar, 1% glucose, 6% fresh yeast, 9.3% molasses, 8.4% coarse semolina, 0.9% acid mix, and 1.7% Tegosept). The following *Drosophila* lines were obtained from the Bloomington *Drosophila* Stock Centre (Indiana, USA): w^1118^ (BL3605), *GAD1-GAL4* (BL51630). The *GAD1-GAL4* line (BL51630, 3.089 kb fragment of the *GAD1* promoter) drives *GAL4* expression in GABAergic neurons (Ki and Lim 2019; Ng et al. 2002)*.* The UAS-*mad2*^RNAi^ (VDRC 47918) was obtained from Vienna Drosophila Resource Centre, Austria. The polyalanine repeat sequence stock (GCA_90_) was obtained from Dr Louise O’Keefe’s laboratory at the University of Adelaide (van Eyk et al. 2012). The expression pattern of the *GAD1-GAL4* driver is shown in supplementary Fig-1 (Nassel et al. 2008). In all cases where there is a negative control, it is generated from the cross between Gad1-Gal4 with w^1118^, while the experimental cross is Gad1-Gal4 with UAS-Mad2RNAi.

### Climbing Assays

Climbing assays capitalise on the natural tendency of flies to climb, known as negative geotaxis. Climbing assays were performed according to the previous published methodology with little modification (van Eyk et al. 2012). Climbing assays were performed on flies either wild-type control, *Mad2*-depleted flies, and polyalanine repeat sequence stock (GCA_90_) under the *GAD1-GAL4* driver. For every genotype, three sets of 15 to 20 freshly enclosed male or female flies were gathered and maintained at 25 °C on a fortified medium until the test was carried out. When the flies were analysed, they were all 8–12 days old. Each batch of flies was moved to a 500 ml measuring cylinder with a 48 mm diameter and a parafilm-sealed lid and allowed to acclimatise for 3 min before the cylinder was tapped on the bench to give twenty strong mechanical shocks. A Dino-Lite digital microscope (Product# AD3713TB; AnMo Electronics Corporation, New Taipei City, Taiwan) was used to record and save the videos of all the experiments. Videos were replayed to count the number of flies remaining below the 1 cm mark, between 1- and 5-cm mark, and above the 5-cm mark after 40 s. The polyalanine repeat sequence stock (GCA_90_) was obtained from Dr Louise O’Keefe’s laboratory as a positive control known to be defective in climbing assays (van Eyk et al. 2012). The chi-squared test was used to analyse the proportion of climbing defects between each genotype compared to the wild-type control.

### Bang-Sensitive Behavioural Assays to Investigate a Seizure-Like Phenotype

The bang-sensitivity behavioural assay (banging assay) was used to measure the recovery of *Drosophila* from seizure-like activities induced by mechanical shock and was performed as described previously (Tao et al. 2011). Experiments were performed between 8 and 11 am to minimise the potential effects of circadian oscillation on animal activity. Between ten and twenty *Drosophila* (females and males) aged between 8 and 12 days after eclosion were collected under CO_2_. The *Drosophila* were transferred to an empty clear 500-ml measuring cylinder and allowed to acclimatise for 3 min before the cylinder was tapped on the bench to give twenty strong mechanical shocks. We considered the flies for seizure-like phenotype based on the following characteristics: unusual loss of posture, erratic flapping and buzzing of the wings, leg shaking, spinning, and uncontrollably flying, as well as total immobilisation and falling during clumsy attempts at rising and flying. A Dino-Lite digital microscope (Product# AD3713TB; AnMo Electronics Corporation, New Taipei City, Taiwan) was used to record and save the videos of all the experiments. Videos were replayed to score the *Drosophila* behaviour. *Drosophila* showing seizure-like behaviour were counted for 30 s. The Fisher’s exact test was used to analyse the data between genotypes compared to the wild-type control. The polyalanine repeat sequence stock (GCA_90_) was obtained from Dr Louise O’Keefe’s laboratory as a positive control for seizure assays (van Eyk et al. 2012). 

### Drug Treatments

Drugs were purchased from Sigma. Drugs were mixed with common fly food for larvae (water, molasses, yeast, glucose, acid-mix, agar, semolina, Tegosept), and when the mixture solidified, they were administered to the host animals throughout their life cycle. The antioxidant utilised was N-acetylcysteine amide (NACA, Sigma A0737) 0 µg/ml, 100 µg/ml, 200 µg/ml, and 400 µg/ml.

### Data Analysis

**Fig. 1:**
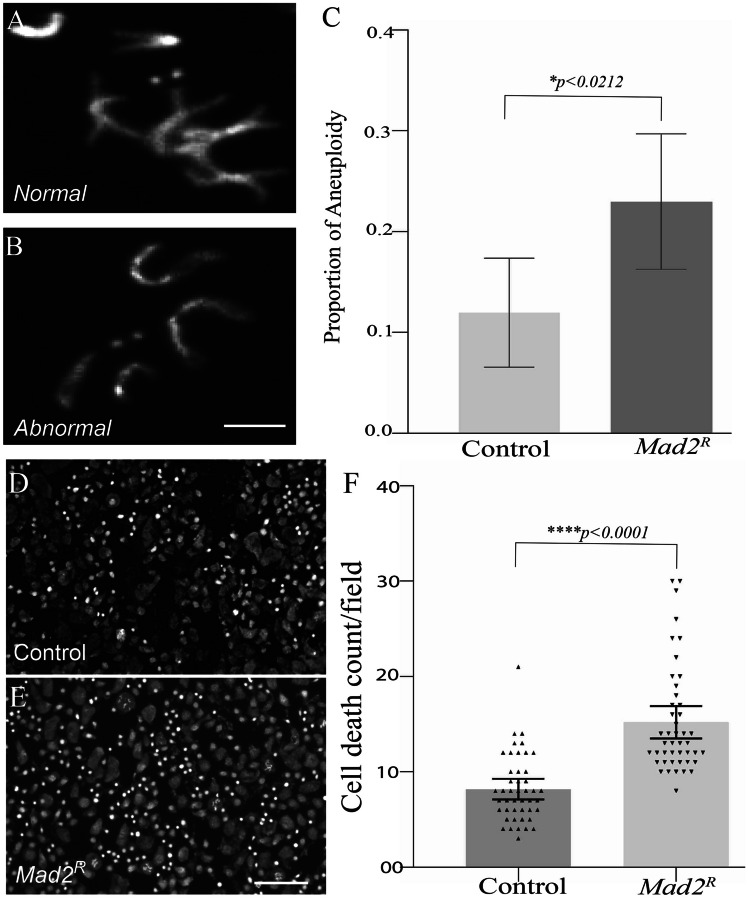
*Mad2* depletion in GABAergic neurons increased the proportion of aneuploidy and cell death. **A** Normal karyotype with four chromosome pairs; **B** abnormal karyotype missing a chromosome pair; and **C** analysis of aneuploidy proportion in wild-type controls and *Mad2*-depleted larval brains. There are aneuploid cells in wild-type brains, but the proportion of karyotypes that were aneuploid significantly increased in *Mad2-*depleted brain cells compared to the control (**C**). The *p*-values were calculated by Fisher’s exact test *p < 0.0212, *n* > 130 karyotypes from ≥ 7 animals for each genotype. The scale bar = 50 μm. Hoechst stain was used to visualise DNA. **D** DNA staining in a wild-type larval brain; **F** DNA staining in a *Mad2*-depleted larval brain; and **E** showing the analysis of pyknotic nuclei in wild-type vs *Mad2*. The *p*-values were calculated by the Mann–Whitney test ****p < 0.0001. The scale bar = 50 μm, *n* = 45 from ≥ 8 animals for each genotype

**Fig. 2:**
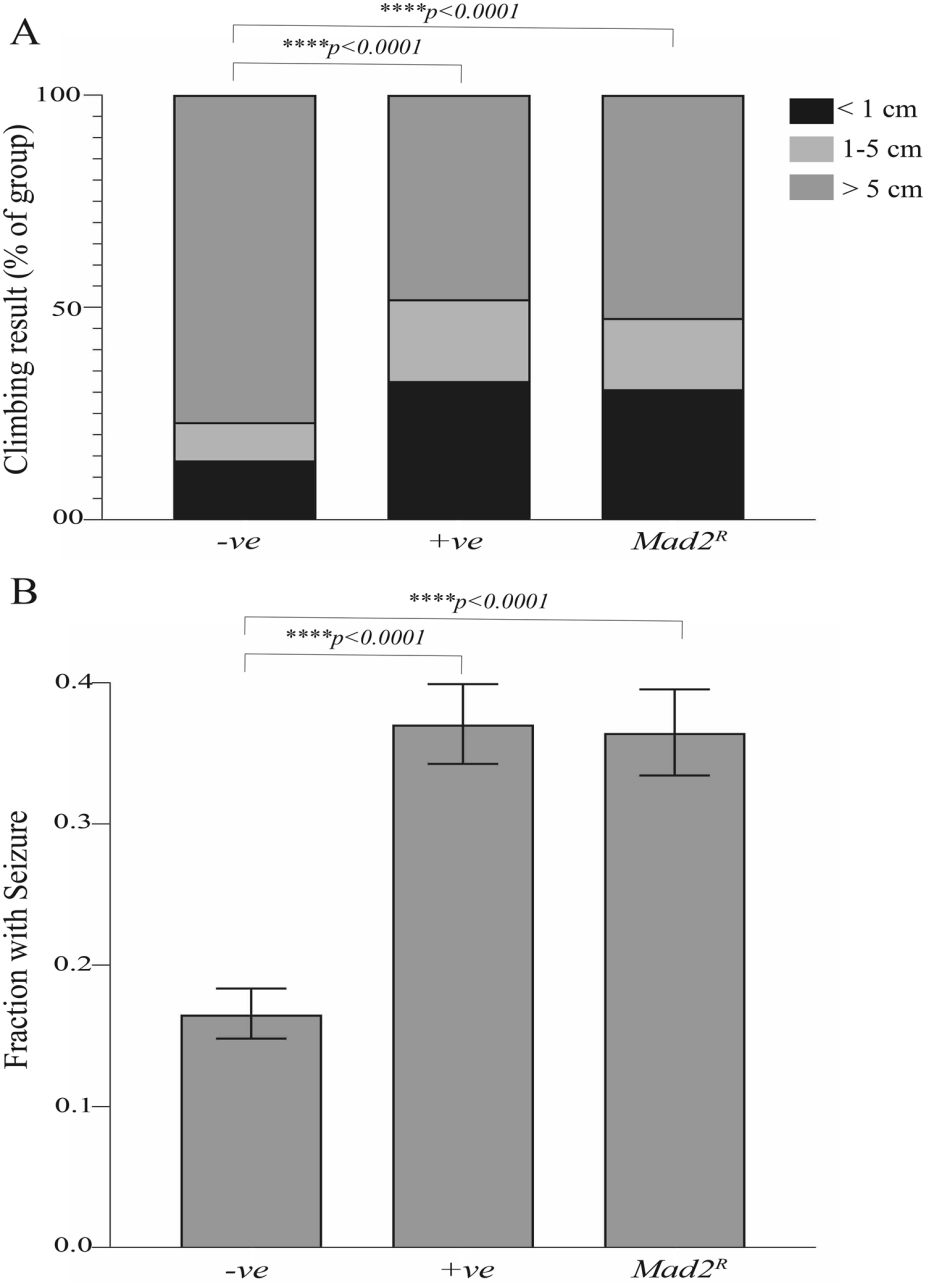
*Mad2* depletion in GABAergic neurons increased the frequency of climbing defects (**A**) and seizure phenotypes (**B**) in flies aged between 8 and 12 days. Flies with *Mad2* depletion in GABAergic neurons showed significant climbing defects compared to the wild-type control (**A**). Positive control: + ve, wild type: − ve. We used overexpression in GABAergic neurons of GCA_90,_ a polyalanine repeat sequence, as a positive control for neurodegenerative climbing defects (van Eyk et al. 2012). The *p*-values were calculated by chi-squared test ****p < 0.0001, *n* > 600 for each genotype. The incidence of seizure-like phenotypes (**B**) significantly increased in *Mad2*-depleted flies compared to the wild-type control flies. The GCA_90_ polyalanine repeat sequence was used as a positive control for seizure (van Eyk et al. 2012). The *p*-values were calculated by Fisher’s exact test ****p < 0.0001, *n* > 950 for each genotype

GraphPad Prism was utilised for statistical analysis. Comparisons of proportions were carried out using Fisher’s exact test where possible; otherwise, chi-squared tests were used (where there were multiple outcomes such as Fig. 2A). Where samples had skewed distributions (e.g. Figure 1F), Mann–Whitney testing was used rather than a *t* test. All error bars indicate 95% confidence intervals for the mean.

## Results

### *Mad2* Depletion in GABAergic Neuron Increased the Aneuploidy Rate in the Larval Brain Which Enhances Cell Death

To investigate the effect of *Mad2* depletion in GABAergic (inhibitory) neurons, we used *Drosophila* as a model, depleting *Mad2* by RNA interference using the *Gad1-Gal4* driver. The knockdown of *Mad2* in GABAergic neurons gave a significant increase in the aneuploidy rate in third-instar larval brains compared to the wild-type control (Fig. 1C). We observed losses of a chromosome pair or one chromosome from the pair. (Fig. 1A, B). This confirmed that we were able to generate the desired modest increase in aneuploidy in the central nervous system. The molecular mechanisms by which aneuploidy can arise in the CNS are a current topic of debate that we have not addressed experimentally (see “Discussion”). Previous published results found that increased aneuploidy enhanced oxidative stress and cell death (Liu et al. 2016; Shaukat et al. 2015; Sheltzer et al. 2012). Different brain pathologies and clinical characteristics are present in age-associated neurodegenerative illnesses, all of which are linked to decreased neuronal numbers in particular brain regions (Wyss-Coray 2016). Therefore, we wanted to see whether enhanced aneuploidy had any detrimental effects on neuronal cells. To measure cell death, we counted pyknotic nuclei in third-instar larval brains. Pyknosis is an irreversible condensation of the DNA usually associated with apoptotic or necrotic cell death. We found that this cell death significantly increased in *Mad2*-depleted larval brains compared to the wild-type control (Fig. 1D, E, F). Because increased aneuploidy enhanced brain cell death in the early stages of development, we then looked for neurological defects, particularly locomotor defects, in adult *Drosophila.*

### *Mad2* Depletion in GABAergic Neurons Caused Neurological Phenotypes

We carried out neurological phenotype analysis based on previous experimental approaches known to detect neurodegeneration. The climbing assay has revealed neurodegenerative defects in the investigation of numerous conditions such as Alzheimer’s disease (Chakraborty et al. 2011). We measured climbing performance in 8- to 12-day-old flies, recording the proportion that was unable to reach the 1-cm mark, those between the 1- and 5-cm mark, and those above the 5-cm mark (normal climbing ability). *Mad2* depletion in GABAergic neurons significantly degraded climbing ability compared to the wild-type control (Fig. 2A), close to the level of functional impairment seen when a well-characterised neurodegeneration was induced by overexpression of a polyalanine repeat sequence (+ ve control) (van Eyk et al. 2012).

We also carried out seizure phenotype analysis in 8- to 12-day-old flies. In *Drosophila*, a seizure-like phenotype can be generated by a range of physical stressors. It is characterised by an unusual loss of posture, erratic flapping and buzzing of the wings, leg shaking, spinning, and uncontrolled flying, as well as total immobilisation and falling during clumsy attempts at rising and flying. The incidence of these phenotypes has been extensively used as a measure of neurological impairment in *Drosophila* models for Alzheimer’s, Huntington’s, and Parkinson’s disease (Holth et al. 2013; Jacquemyn et al. 2023; Lee et al. 2004). *Mad2* depletion in GABAergic neurons significantly increased the incidence of seizure-like phenotypes compared to the wild-type control (Fig. 2B), reaching a level comparable with the repeat sequence positive control.

Because male *Drosophila* are dosage-compensated aneuploids relative to females, we tested whether there was any effect of sex on these *Mad2*-induced climbing and seizure phenotypes. We performed these analyses separately for males and females and found no significant difference between the sexes in climbing defects or in seizure phenotypes (Supplementary Fig-2A, 2B).

### Antioxidant Feeding Rescued the Climbing Defect and Seizure Phenotype in Adult Drosophila

Oxidative stress is a typical cellular response to aneuploidy (Liu et al. 2016; Shaukat et al. 2015), and in some cases, feeding antioxidants can rescue the cell death caused by aneuploidy (Islam et al. 2023; Liu et al. 2016). NACA is an effective antioxidant in *Drosophila*, and one of the benefits of NACA feeding is that it can pass the blood–brain barrier (Moulton et al. 2021).

**Fig. 3:**
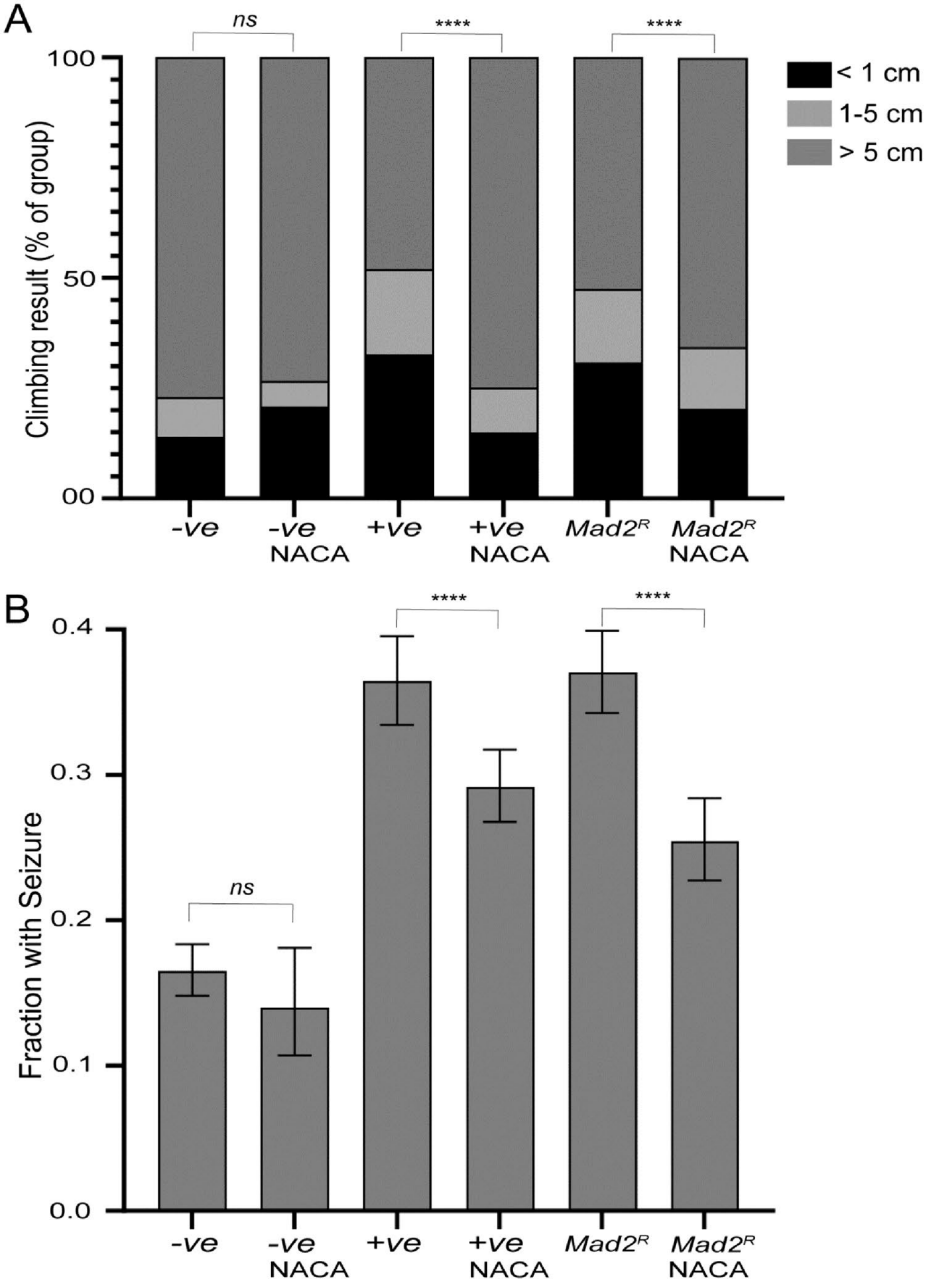
Antioxidant feeding rescues the climbing defects (**A**) and seizure phenotype (**B**) in aneuploid flies aged between 8 and 12 days. 100 µg/ml N-acetyl cysteine amide (NACA) feeding significantly decreased the climbing defects compared to the positive control (+ ve) and *Mad2*-depleted flies (**A**). The *p*-values were calculated by Fisher’s exact test comparing proportions climbing less than 5 cm ****p < 0.0001, *ns*: *p* > 0.05, *n* > 450 for each genotype. The GCA_90_ polyalanine repeat sequence was used as a positive control for climbing defects and seizure phenotype. Seizure phenotype (**B**) significantly increased in *Mad2*-depleted flies compared to the wild type of control flies which were rescued by 100 µg/ml NACA feeding for both + ve (positive control) and *Mad2*-depleted flies. The *p*-values were calculated by Fisher’s exact test ****p < 0.0001, *ns*: *p* > 0.05, *n* > 300 for each genotype

NACA feeding showed a significant reduction of climbing defects (Fig. 3A) as well as a significant reduction of seizure phenotypes (Fig. 3B) in the flies. In our assays, even 100 µg/ml NACA feeding was sufficient to show rescue of the neurological phenotypes, with no advantage from higher doses (Supplementary Fig-2C, 2D). The rescue was not complete, suggesting that although oxidative stress is clearly a major part of the aneuploidy phenotype, there are likely to be other contributing factors (such as protein folding stress, etc.) (Haynes et al. 2004; Khan et al. 2018). The GCA_90_, polyalanine repeat sequence was used as a positive control for climbing defects and seizure phenotypes (van Eyk et al. 2012), and as expected, it was also significantly rescued by antioxidant feeding.

## Discussion

The role of aneuploidy in the pathogenesis of various diseases, particularly in neurological disorders, has been a subject of intense investigation. In this study, we explored the link between increased aneuploidy and the development of a neurological phenotype, focusing on the impact of *Mad2* depletion in the *Drosophila* model. Our findings provide compelling evidence that aneuploidy, induced by the depletion of *Mad2*, leads to oxidative stress and subsequently contributes to the manifestation of functional neurological deficits.

We found that *Mad2* depletion in third-instar larval brains leads to increased aneuploidy, consistent with previous studies that have identified a role for *Mad2* in neurological disease progression (Shi et al. 2019; Wu et al. 2018). Our findings align with studies in mammals, where aneuploidy has been implicated in neurodevelopmental disorders, including intellectual disabilities and autism spectrum disorders (Iourov et al. 2006). One of the most common causes of dementia among the aging population has implicated aneuploidy: Alzheimer’s disease. Lymphocytes and splenocytes isolated from AD patients show mitosis and chromosomal segregation defects (Migliore et al. 1999; Petrozzi et al. 2002), demonstrating AD patients’ vulnerability to aneuploidy and their predisposition to produce aneuploid cells. In some studies, as much as 90% of the cell death observed in AD neurons has been attributed to aneuploid neurons (Arendt et al. 2010).

In our current study, we present evidence suggesting that increased aneuploidy leads to more cell death in the brain. Consistent with this, there is evidence that microcephaly is caused by *KNL1* mutations. Like *Mad2*, *KNL1* mediates the spindle assembly checkpoint, which acts as a protective mechanism against aneuploidy. Segregation mistakes in mitotic neural progenitor cells subsequent to *KNL1* deletion result in DNA damage on the missegregated chromosomes. This leads to a large-scale eradication of cells with somatic genome damage by p53 activation and strong apoptotic and microglial phagocytic responses, ultimately resulting in microcephaly (Shi et al. 2019). In several other experimental systems, aneuploidy has been shown to promote cell death like neurodegeneration (Kai et al. 2009; Oromendia and Amon 2014; Rajendran et al. 2008) consistent with our current study. Previously, we have shown that *Mad2* depletion causes chromosomal instability which generates aneuploid cells (Shaukat et al. 2012), resulting in mitochondrial stress, proteotoxic stress, DNA damage, alteration of cellular signalling pathways, and cell death in proliferating *Drosophila* epithelia (Islam et al. 2023; Khan et al. 2018; Liu et al. 2016; Shaukat et al. 2015). In these cases, cell death has been apoptotic, but we have not yet confirmed whether neuronal cell death in response to aneuploidy is also apoptotic. It would be desirable to validate our model of aneuploidy using alternative methods to deplete Mad2. We have previously used null mutants, but in this case, the effect of systemic aneuploidy would make the interpretation of behavioural phenotypes highly problematic. The advantage of the model we use here is that it is targeted to a very narrow subset of brain cells, so we can be confident that they are responsible for the behavioural phenotypes observed.

Since neurons have historically been believed to remain post-mitotic (Bhardwaj et al. 2006), how mosaic aneuploidy can occur in neurodegenerative or neurodevelopmental disorders has been unclear. Aneuploidy rates in the hippocampus, cortex, and cerebellum of the brain were studied by Iourov and co-workers, relating AD patients with a group of unaffected aged-matched controls (Iourov et al. 2008; Yurov et al. 2007). They suggested that the increased levels of aneuploidy in AD were due to aberrant adult neurogenesis resulting from mitotic non-disjunction in neurons re-entering the cell cycle. Neurogenesis in the adult brain may be an indicator for the initial identification of AD (Culig et al. 2022). The main evidence for this hypothesis is that neurons of the AD brain showed cyclin B1, cyclin D1, cdc2, and Ki67 phospho-protein expression which are normally identified during mitosis (Arendt 2012; McShea et al. 1997; Vincent et al. 1997, 1996; Yang et al. 2001). Additionally, it has been demonstrated that amyloid beta peptide (Aβ) triggers the expression of mitotic proteins and cell cycle entrance by mature neurons in culture (Absalon et al. 2013; Majd et al. 2008; Seward et al. 2013). This raises the possibility that re-entering into an aberrant cell cycle may result in aneuploidy. Previously, aneuploidy was presumed to result from proliferation during development, with defective clearance of these cells explaining their existence in the adult brain (Devalle et al. 2012; Rohrback et al. 2018). In recent times, it has become clear that neurogenesis is more common than had been assumed and that the potential for neurogenesis persists into old age, even though it is not usually used (Boldrini et al. 2018; Sorrells et al. 2018; Spalding et al. 2013; Zhao et al. 2008). There is good evidence that neurogenesis can arise in many areas of the brain during life. In general, genetic and environmental stressors can produce and accumulate aneuploidy in the division or regeneration of cell populations at any time in life (Oromendia and Amon 2014; Potter 1991). In addition, evidence from several studies indicates that neurogenesis in many brain regions can be induced in adult mice and rats in response to brain injury and attempted self-repair by the brain (Ibrahim et al. 2016; Zheng et al. 2013; Zhou et al. 2004).

Recent evidence has shown that striatal astrocytes may transdifferentiate into new neurons able to form active neuronal circuits with pre-existing neurons following ischemic brain injury, which is the basis of an alternative potential mechanism for neuronal aneuploidy (Zheng et al. 2013; Zhou et al. 2004). These results suggest that in AD and FTLD-MAPT brains, some of the aneuploid neurons may originate from glia.

Increased total exposure to environmental stressors is related to aging, which can enhance the missegregation of chromosomes and neuronal aneuploidy (Iourov et al. 2013; Potter 1991). Age may be triggering all other mechanisms mentioned to form neuronal aneuploidy because evidence has shown that neuronal and non-neuronal aneuploidy increase with age (Arendt et al. 2009; Fantin et al. 2019; Fischer et al. 2012; Yurov et al. 2009, 2010). It appears, then, that there are several possible mechanisms to account for the observed aneuploidy in adult neurons, which our experiments have been modeling with the objective of identifying potential interventions. We do not know which, if any, of these mechanisms are contributing to the aneuploidy generated in our model. Because we are using Mad2 depletion, it is likely that adult cell division (rather than fusion) is involved. Previous experiments to induce neuronal aneuploidy, such as by expressing mutant Tau protein, have shown similar neuronal cell death (Caneus et al. 2018), though in that case, because Tau has many effects, it was harder to confidently attribute the phenotype to aneuploidy.

We identified oxidative stress as a significant mediator of the neurological phenotype observed in *Mad2*-depleted *Drosophila*. Oxidative stress is a stereotypical cellular response to aneuploidy (Newman and Gregory 2019), and oxidative stress is also known to be a common detrimental observation in various neurodevelopmental disorders and neurodegenerative diseases (Barnham et al. 2004). Our data suggested that induced aneuploidy in GABAergic neurons increased oxidative stress and generated functional neurological phenotypes. Feeding antioxidants could rescue the neurological defects, showing that oxidative stress was a significant contributor to the neurological phenotype generated by aneuploidy. Reactive oxygen species (ROS) production typically leads to protein oxidation, lipid peroxidation, and DNA damage, all of which are implicated in the pathogenesis of neurodegenerative diseases (Singh et al. 2019). The antioxidant we used, NACA, is known to significantly reduce the accumulation of lipid droplets (LD) and the production of peroxidised lipids in flies (Moulton et al. 2021). LD buildup in the brain is triggered by increased oxidative stress, and the dysregulation of lipid droplets is known to play a role in the progression of neurological diseases (Farmer et al. 2020). The known associations between oxidative stress, neurodegeneration, and aging suggest a likely effect of neuronal aneuploidy on lifespan, which would be worth testing in the future.

In conclusion, our study provides important insights into the connection between increased aneuploidy and the development of a neurological phenotype in a *Drosophila* model system. We highlight the role of oxidative stress as a mediator of this phenotype and suggest potential therapeutic avenues for neurodegenerative diseases. This research contributes to our understanding of the complex interplay between aneuploidy and neurobiology and opens new doors for further investigations into the pathogenesis and treatment of neurological disorders.

### Supplementary Information

Below is the link to the electronic supplementary material.

Supplementary file1 Fig-1: The expression pattern of Gad1-Gal4 driver visualised by CD8-GFP in a 3rd instar Drosophila larval central nervous system.(TIF 12292 KB)

Supplementary file2 Fig-2: The climbing performance and seizure-like phenotype in male vs females are shown in A (each group shown, avg >30 individuals per group) and B (n>270 for each genotype). No significant difference between male vs females were detected regarding the climbing performance and seizure like phenotypes. The effect of various doses of NACA feeding on Gad1-Gal4, UAS-Mad2-RNAi induced climbing defects and seizure like phenotypes are shown in C (n>230 for each dosage) and D (n>280 for each dosage). There was no significant improvement above 100 ug/ml for rescuing the climbing defects and seizure like-phenotype (C, D).(TIF 737 KB)

## Data Availability

No datasets were generated or analyzed during the current study.

## References

[CR1] Absalon S, Kochanek DM, Raghavan V, Krichevsky AM (2013) MiR-26b, upregulated in Alzheimer’s disease, activates cell cycle entry, tau-phosphorylation, and apoptosis in postmitotic neurons. J Neurosci 33(37):14645–14659. 10.1523/JNEUROSCI.1327-13.2013 24027266 10.1523/JNEUROSCI.1327-13.2013 PMC3810537

[CR2] Arendt T (2012) Cell cycle activation and aneuploid neurons in Alzheimer’s disease. Mol Neurobiol 46(1):125–135. 10.1007/s12035-012-8262-0 22528601 10.1007/s12035-012-8262-0

[CR3] Arendt T, Mosch B, Morawski M (2009) Neuronal aneuploidy in health and disease: a cytomic approach to understand the molecular individuality of neurons. Int J Mol Sci 10(4):1609–1627. 10.3390/ijms10041609 19468329 10.3390/ijms10041609 PMC2680637

[CR4] Arendt T, Bruckner MK, Mosch B, Losche A (2010) Selective cell death of hyperploid neurons in Alzheimer’s disease. Am J Pathol 177(1):15–20. 10.2353/ajpath.2010.090955 20472889 10.2353/ajpath.2010.090955 PMC2893646

[CR5] Arendt T, Stieler J, Ueberham U (2017) Is sporadic Alzheimer’s disease a developmental disorder? J Neurochem 143(4):396–408. 10.1111/jnc.14036 28397252 10.1111/jnc.14036

[CR6] Bajic G, Degn SE, Thiel S, Andersen GR (2015) Complement activation, regulation, and molecular basis for complement-related diseases. EMBO J 34(2):2735–2757. 10.15252/embj.201591881 26489954 10.15252/embj.201591881 PMC4682646

[CR7] Barnham KJ, Masters CL, Bush AI (2004) Neurodegenerative diseases and oxidative stress. Nat Rev Drug Discov 3(3):205–214. 10.1038/nrd1330 15031734 10.1038/nrd1330

[CR8] Bhardwaj RD, Curtis MA, Spalding KL, Buchholz BA, Fink D, Bjork-Eriksson T, Nordborg C, Gage FH, Druid H, Eriksson PS, Frisen J (2006) Neocortical neurogenesis in humans is restricted to development. Proc Natl Acad Sci U S A 103(33):12564–12568. 10.1073/pnas.0605177103 16901981 10.1073/pnas.0605177103 PMC1567918

[CR9] Boeras DI, Granic A, Padmanabhan J, Crespo NC, Rojiani AM, Potter H (2008) Alzheimer’s presenilin 1 causes chromosome missegregation and aneuploidy. Neurobiol Aging 29(3):319–328. 10.1016/j.neurobiolaging.2006.10.027 17169464 10.1016/j.neurobiolaging.2006.10.027 PMC2692942

[CR10] Boldrini M, Fulmore CA, Tartt AN, Simeon LR, Pavlova I, Poposka V, Rosoklija GB, Stankov A, Arango V, Dwork AJ, Hen R, Mann JJ (2018) Human hippocampal neurogenesis persists throughout aging. Cell Stem Cell 22(4):589-599 e585. 10.1016/j.stem.2018.03.015 29625071 10.1016/j.stem.2018.03.015 PMC5957089

[CR11] Buckton KE, Whalley LJ, Lee M, Christie JE (1982) Chromosome aneuploidy in Alzheimer’s disease. Exp Brain Res, Suppl 5:58–63. 10.1007/978-3-642-68507-1_9 10.1007/978-3-642-68507-1_9 7151922

[CR12] Buckton KE, Whalley LJ, Lee M, Christie JE (1983) Chromosome changes in Alzheimer’s presenile dementia. J Med Genet 20(1):46–51. 10.1136/jmg.20.1.46 6842534 10.1136/jmg.20.1.46 PMC1048985

[CR13] Buffin E, Emre D, Karess RE (2007) Flies without a spindle checkpoint. Nat Cell Biol 9(5):565–572. 10.1038/ncb1570 17417628 10.1038/ncb1570

[CR14] Caneus J, Granic A, Rademakers R, Dickson DW, Coughlan CM, Chial HJ, Potter H (2018) Mitotic defects lead to neuronal aneuploidy and apoptosis in frontotemporal lobar degeneration caused by MAPT mutations. Mol Biol Cell 29(5):575–586. 10.1091/mbc.E17-01-0031 29282277 10.1091/mbc.E17-01-0031 PMC6004587

[CR15] Chakraborty R, Vepuri V, Mhatre SD, Paddock BE, Miller S, Michelson SJ, Delvadia R, Desai A, Vinokur M, Melicharek DJ, Utreja S, Khandelwal P, Ansaloni S, Goldstein LE, Moir RD, Lee JC, Tabb LP, Saunders AJ, Marenda DR (2011) Characterization of a Drosophila Alzheimer’s disease model: pharmacological rescue of cognitive defects. PLoS ONE 6(6):e20799. 10.1371/journal.pone.0020799 21673973 10.1371/journal.pone.0020799 PMC3108982

[CR16] Culig L, Chu X, Bohr VA (2022) Neurogenesis in aging and age-related neurodegenerative diseases. Ageing Res Rev 78:101636. 10.1016/j.arr.2022.101636 35490966 10.1016/j.arr.2022.101636 PMC9168971

[CR17] Devalle S, Sartore RC, Paulsen BS, Borges HL, Martins RA, Rehen SK (2012) Implications of aneuploidy for stem cell biology and brain therapeutics. Front Cell Neurosci 6:36. 10.3389/fncel.2012.00036 22973193 10.3389/fncel.2012.00036 PMC3433681

[CR18] Faggioli F, Vijg J, Montagna C (2011) Chromosomal aneuploidy in the aging brain. Mech Ageing Dev 132(8–9):429–436. 10.1016/j.mad.2011.04.008 21549743 10.1016/j.mad.2011.04.008 PMC3168579

[CR19] Fantin F, Macchi F, Giani A, Bissoli L (2019) The importance of nutrition in hypertension. Nutrients 11(10). 10.3390/nu11102542 10.3390/nu11102542 PMC6835472 31640287

[CR20] Farmer BC, Walsh AE, Kluemper JC, Johnson LA (2020) Lipid droplets in neurodegenerative disorders. Front Neurosci 14:742. 10.3389/fnins.2020.00742 32848541 10.3389/fnins.2020.00742 PMC7403481

[CR21] Fischer HG, Morawski M, Bruckner MK, Mittag A, Tarnok A, Arendt T (2012) Changes in neuronal DNA content variation in the human brain during aging. Aging Cell 11(4):628–633. 10.1111/j.1474-9726.2012.00826.x 22510449 10.1111/j.1474-9726.2012.00826.x

[CR22] Geller LN, Potter H (1999) Chromosome missegregation and trisomy 21 mosaicism in Alzheimer’s disease. Neurobiol Dis 6(3):167–179. 10.1006/nbdi.1999.0236 10408806 10.1006/nbdi.1999.0236

[CR23] Granic A, Padmanabhan J, Norden M, Potter H (2010) Alzheimer Abeta peptide induces chromosome mis-segregation and aneuploidy, including trisomy 21: requirement for tau and APP. Mol Biol Cell 21(4):511–520. 10.1091/mbc.E09-10-0850 20032300 10.1091/mbc.E09-10-0850 PMC2820417

[CR24] Haynes CM, Titus EA, Cooper AA (2004) Degradation of misfolded proteins prevents ER-derived oxidative stress and cell death. Mol Cell 15(5):767–776. 10.1016/j.molcel.2004.08.025 15350220 10.1016/j.molcel.2004.08.025

[CR25] Holth JK, Bomben VC, Reed JG, Inoue T, Younkin L, Younkin SG, Pautler RG, Botas J, Noebels JL (2013) Tau loss attenuates neuronal network hyperexcitability in mouse and Drosophila genetic models of epilepsy. J Neurosci 33(4):1651–1659. 10.1523/JNEUROSCI.3191-12.2013 23345237 10.1523/JNEUROSCI.3191-12.2013 PMC3711605

[CR26] Ibrahim S, Hu W, Wang X, Gao X, He C, Chen J (2016) Traumatic brain injury causes aberrant migration of adult-born neurons in the hippocampus. Sci Rep 6:21793. 10.1038/srep21793 26898165 10.1038/srep21793 PMC4761898

[CR27] Iourov IY, Vorsanova SG, Yurov YB (2006) Chromosomal variation in mammalian neuronal cells: known facts and attractive hypotheses. Int Rev Cytol 249:143–191. 10.1016/S0074-7696(06)49003-3 16697283 10.1016/S0074-7696(06)49003-3

[CR28] Iourov IY, Vorsanova SG, Yurov YB (2008) Chromosomal mosaicism goes global. Mol Cytogenet 1:26. 10.1186/1755-8166-1-26 19032785 10.1186/1755-8166-1-26 PMC2612668

[CR29] Iourov IY, Vorsanova SG, Liehr T, Kolotii AD, Yurov YB (2009) Increased chromosome instability dramatically disrupts neural genome integrity and mediates cerebellar degeneration in the ataxia-telangiectasia brain. Hum Mol Genet 18(14):2656–2669. 10.1093/hmg/ddp207 19414482 10.1093/hmg/ddp207

[CR30] Iourov IY, Vorsanova SG, Yurov YB (2013) Somatic cell genomics of brain disorders: a new opportunity to clarify genetic-environmental interactions. Cytogenet Genome Res 139(3):181–188. 10.1159/000347053 23428498 10.1159/000347053

[CR31] Islam A, Shaukat Z, Newman DL, Hussain R, Ricos MG, Dibbens L, Gregory SL (2023) Chromosomal instability causes sensitivity to polyamines and one-carbon metabolism. Metabolites 13(5). 10.3390/metabo13050642 10.3390/metabo13050642 PMC10221085 37233683

[CR32] Jacquemyn J, Kuenen S, Swerts J, Pavie B, Vijayan V, Kilic A, Chabot D, Wang YC, Schoovaerts N, Corthout N, Verstreken P (2023) Parkinsonism mutations in DNAJC6 cause lipid defects and neurodegeneration that are rescued by Synj1. NPJ Parkinsons Dis 9(1):19. 10.1038/s41531-023-00459-3 36739293 10.1038/s41531-023-00459-3 PMC9899244

[CR33] Kai Y, Wang CC, Kishigami S, Kazuki Y, Abe S, Takiguchi M, Shirayoshi Y, Inoue T, Ito H, Wakayama T, Oshimura M (2009) Enhanced apoptosis during early neuronal differentiation in mouse ES cells with autosomal imbalance. Cell Res 19(2):247–258. 10.1038/cr.2008.305 19015669 10.1038/cr.2008.305

[CR34] Khan M, Shaukat Z, Saint R, Gregory SL (2018) Chromosomal instability causes sensitivity to protein folding stress and ATP depletion. Biol Open 7(10). 10.1242/bio.038000 10.1242/bio.038000 PMC6215417 30327366

[CR35] Ki Y, Lim C (2019) Sleep-promoting effects of threonine link amino acid metabolism in Drosophila neuron to GABAergic control of sleep drive. Elife 8. 10.7554/eLife.40593 10.7554/eLife.40593 PMC6636906 31313987

[CR36] Kingsbury MA, Friedman B, McConnell MJ, Rehen SK, Yang AH, Kaushal D, Chun J (2005) Aneuploid neurons are functionally active and integrated into brain circuitry. Proc Natl Acad Sci U S A 102(17):6143–6147. 10.1073/pnas.0408171102 15837924 10.1073/pnas.0408171102 PMC1087909

[CR37] Kleppner SR, Tobin AJ (2001) GABA signalling: therapeutic targets for epilepsy, Parkinson’s disease and Huntington’s disease. Expert Opin Ther Targets 5(2):219–239. 10.1517/14728222.5.2.219 15992178 10.1517/14728222.5.2.219

[CR38] Lee WC, Yoshihara M, Littleton JT (2004) Cytoplasmic aggregates trap polyglutamine-containing proteins and block axonal transport in a Drosophila model of Huntington’s disease. Proc Natl Acad Sci U S A 101(9):3224–3229. 10.1073/pnas.0400243101 14978262 10.1073/pnas.0400243101 PMC365771

[CR39] Lentini L, Barra V, Schillaci T, Di Leonardo A (2012) MAD2 depletion triggers premature cellular senescence in human primary fibroblasts by activating a p53 pathway preventing aneuploid cells propagation. J Cell Physiol 227(9):3324–3332. 10.1002/jcp.24030 22170163 10.1002/jcp.24030

[CR40] Liu D, Shaukat Z, Xu T, Denton D, Saint R, Gregory S (2016) Autophagy regulates the survival of cells with chromosomal instability. Oncotarget 7(39):63913–63923. 10.18632/oncotarget.11736 27590505 10.18632/oncotarget.11736 PMC5325413

[CR41] Majd S, Zarifkar A, Rastegar K, Takhshid MA (2008) Different fibrillar Abeta 1–42 concentrations induce adult hippocampal neurons to reenter various phases of the cell cycle. Brain Res 1218:224–229. 10.1016/j.brainres.2008.04.050 18533137 10.1016/j.brainres.2008.04.050

[CR42] Matsuyama SS, Bohman R (1988) Variation in DNA content of mononuclear cells of patients with dementia of the Alzheimer type. Alzheimer Dis Assoc Disord 2(2):120–122. 10.1097/00002093-198802020-00004 3214580 10.1097/00002093-198802020-00004

[CR43] McShea A, Harris PL, Webster KR, Wahl AF, Smith MA (1997) Abnormal expression of the cell cycle regulators P16 and CDK4 in Alzheimer’s disease. Am J Pathol 150(6):1933–1939. https://www.ncbi.nlm.nih.gov/pubmed/9176387 PMC1858317 9176387

[CR44] Migliore L, Botto N, Scarpato R, Petrozzi L, Cipriani G, Bonuccelli U (1999) Preferential occurrence of chromosome 21 malsegregation in peripheral blood lymphocytes of Alzheimer disease patients. Cytogenet Cell Genet 87(1–2):41–46. 10.1159/000015389 10640809 10.1159/000015389

[CR45] Mirkovic M, Guilgur LG, Tavares A, Passagem-Santos D, Oliveira RA (2019) Induced aneuploidy in neural stem cells triggers a delayed stress response and impairs adult life span in flies. PLoS Biol 17(2):e3000016. 10.1371/journal.pbio.3000016 30794535 10.1371/journal.pbio.3000016 PMC6402706

[CR46] Moulton MJ, Barish S, Ralhan I, Chang J, Goodman LD, Harland JG, Marcogliese PC, Johansson JO, Ioannou MS, Bellen HJ (2021) Neuronal ROS-induced glial lipid droplet formation is altered by loss of Alzheimer’s disease-associated genes. Proc Natl Acad Sci U S A 118(52). 10.1073/pnas.2112095118 10.1073/pnas.2112095118 PMC8719885 34949639

[CR47] Nassel DR, Enell LE, Santos JG, Wegener C, Johard HA (2008) A large population of diverse neurons in the Drosophila central nervous system expresses short neuropeptide F, suggesting multiple distributed peptide functions. BMC Neurosci 9:90. 10.1186/1471-2202-9-90 18803813 10.1186/1471-2202-9-90 PMC2569041

[CR48] Newman DL, Gregory SL (2019) Co-operation between aneuploidy and metabolic changes in driving tumorigenesis. Int J Mol Sci 20(18). 10.3390/ijms20184611 10.3390/ijms20184611 PMC6770258 31540349

[CR49] Ng M, Roorda RD, Lima SQ, Zemelman BV, Morcillo P, Miesenbock G (2002) Transmission of olfactory information between three populations of neurons in the antennal lobe of the fly. Neuron 36(3):463–474. 10.1016/s0896-6273(02)00975-3 12408848 10.1016/s0896-6273(02)00975-3

[CR50] Oromendia AB, Amon A (2014) Aneuploidy: implications for protein homeostasis and disease. Dis Model Mech 7(1):15–20. 10.1242/dmm.013391 24396150 10.1242/dmm.013391 PMC3882044

[CR51] Petrozzi L, Lucetti C, Scarpato R, Gambaccini G, Trippi F, Bernardini S, Del Dotto P, Migliore L, Bonuccelli U (2002) Cytogenetic alterations in lymphocytes of Alzheimer’s disease and Parkinson’s disease patients. Neurol Sci 23(Suppl 2):S97-98. 10.1007/s100720200087 12548361 10.1007/s100720200087

[CR52] Potter H (1991) Review and hypothesis: Alzheimer disease and Down syndrome--chromosome 21 nondisjunction may underlie both disorders. Am J Hum Genet 48(6):1192–1200. https://www.ncbi.nlm.nih.gov/pubmed/1827946 PMC1683102 1827946

[CR53] Rajendran RS, Wellbrock UM, Zupanc GK (2008) Apoptotic cell death, long-term persistence, and neuronal differentiation of aneuploid cells generated in the adult brain of teleost fish. Dev Neurobiol 68(10):1257–1268. 10.1002/dneu.20656 18563701 10.1002/dneu.20656

[CR54] Rohrback S, Siddoway B, Liu CS, Chun J (2018) Genomic mosaicism in the developing and adult brain. Dev Neurobiol 78(11):1026–1048. 10.1002/dneu.22626 30027562 10.1002/dneu.22626 PMC6214721

[CR55] Santaguida S, Amon A (2015) Short- and long-term effects of chromosome mis-segregation and aneuploidy. Nat Rev Mol Cell Biol 16(8):473–485. 10.1038/nrm4025 26204159 10.1038/nrm4025

[CR56] Seward ME, Swanson E, Norambuena A, Reimann A, Cochran JN, Li R, Roberson ED, Bloom GS (2013) Amyloid-beta signals through tau to drive ectopic neuronal cell cycle re-entry in Alzheimer’s disease. J Cell Sci 126(Pt 5):1278–1286. 10.1242/jcs.1125880 23345405 10.1242/jcs.1125880 PMC3635465

[CR57] Shaukat Z, Wong HW, Nicolson S, Saint RB, Gregory SL (2012) A screen for selective killing of cells with chromosomal instability induced by a spindle checkpoint defect. PLoS ONE 7(10):e47447. 10.1371/journal.pone.0047447 23077619 10.1371/journal.pone.0047447 PMC3471812

[CR58] Shaukat Z, Liu D, Choo A, Hussain R, O’Keefe L, Richards R, Saint R, Gregory SL (2015) Chromosomal instability causes sensitivity to metabolic stress. Oncogene 34(31):4044–4055. 10.1038/onc.2014.344 25347746 10.1038/onc.2014.344

[CR59] Sheltzer JM, Torres EM, Dunham MJ, Amon A (2012) Transcriptional consequences of aneuploidy. Proc Natl Acad Sci U S A 109(31):12644–12649. 10.1073/pnas.1209227109 22802626 10.1073/pnas.1209227109 PMC3411958

[CR60] Shi L, Qalieh A, Lam MM, Keil JM, Kwan KY (2019) Robust elimination of genome-damaged cells safeguards against brain somatic aneuploidy following Knl1 deletion. Nat Commun 10(1):2588. 10.1038/s41467-019-10411-w 31197172 10.1038/s41467-019-10411-w PMC6565622

[CR61] Singh A, Kukreti R, Saso L, Kukreti S (2019). Oxidative stress: a key modulator in neurodegenerative diseases. Molecules 24(8). 10.3390/molecules24081583 10.3390/molecules24081583 PMC6514564 31013638

[CR62] Sorrells SF, Paredes MF, Cebrian-Silla A, Sandoval K, Qi D, Kelley KW, James D, Mayer S, Chang J, Auguste KI, Chang EF, Gutierrez AJ, Kriegstein AR, Mathern GW, Oldham MC, Huang EJ, Garcia-Verdugo JM, Yang Z, Alvarez-Buylla A (2018) Human hippocampal neurogenesis drops sharply in children to undetectable levels in adults. Nature 555(7696):377–381. 10.1038/nature25975 29513649 10.1038/nature25975 PMC6179355

[CR63] Spalding KL, Bergmann O, Alkass K, Bernard S, Salehpour M, Huttner HB, Bostrom E, Westerlund I, Vial C, Buchholz BA, Possnert G, Mash DC, Druid H, Frisen J (2013) Dynamics of hippocampal neurogenesis in adult humans. Cell 153(6):1219–1227. 10.1016/j.cell.2013.05.002 23746839 10.1016/j.cell.2013.05.002 PMC4394608

[CR64] Tao H, Manak JR, Sowers L, Mei X, Kiyonari H, Abe T, Dahdaleh NS, Yang T, Wu S, Chen S, Fox MH, Gurnett C, Montine T, Bird T, Shaffer LG, Rosenfeld JA, McConnell J, Madan-Khetarpal S, Berry-Kravis E, Griesbach H, Saneto RP, Scott MP, Antic D, Reed J, Boland R, Ehaideb SN, El-Shanti H, Mahajan VB, Ferguson PJ, Axelrod JD, Lehesjoki AE, Fritzsch B, Slusarski DC, Wemmie J, Ueno N, Bassuk AG (2011) Mutations in prickle orthologs cause seizures in flies, mice, and humans. Am J Hum Genet 88(2):138–149. 10.1016/j.ajhg.2010.12.012 21276947 10.1016/j.ajhg.2010.12.012 PMC3035715

[CR65] van Eyk CL, McLeod CJ, O’Keefe LV, Richards RI (2012) Comparative toxicity of polyglutamine, polyalanine and polyleucine tracts in Drosophila models of expanded repeat disease. Hum Mol Genet 21(3):536–547. 10.1093/hmg/ddr487 22021427 10.1093/hmg/ddr487

[CR66] Vincent I, Rosado M, Davies P (1996) Mitotic mechanisms in Alzheimer’s disease? J Cell Biol 132(3):413–425. 10.1083/jcb.132.3.413 8636218 10.1083/jcb.132.3.413 PMC2120731

[CR67] Vincent I, Jicha G, Rosado M, Dickson DW (1997) Aberrant expression of mitotic cdc2/cyclin B1 kinase in degenerating neurons of Alzheimer’s disease brain. J Neurosci 17(10):3588–3598. 10.1523/JNEUROSCI.17-10-03588.1997 9133382 10.1523/JNEUROSCI.17-10-03588.1997 PMC6573674

[CR68] Ward BE, Cook RH, Robinson A, Austin JH (1979) Increased aneuploidy in Alzheimer disease. Am J Med Genet 3(2):137–144. 10.1002/ajmg.1320030204 474626 10.1002/ajmg.1320030204

[CR69] Wu D, Wang L, Yang Y, Huang J, Hu Y, Shu Y, Zhang J, Zheng J (2018) MAD2-p31(comet) axis deficiency reduces cell proliferation, migration and sensitivity of microtubule-interfering agents in glioma. Biochem Biophys Res Commun 498(1):157–163. 10.1016/j.bbrc.2018.02.011 29408509 10.1016/j.bbrc.2018.02.011

[CR70] Wyss-Coray T (2016) Ageing, neurodegeneration and brain rejuvenation. Nature 539(7628):180–186. 10.1038/nature20411 27830812 10.1038/nature20411 PMC5172605

[CR71] Yang Y, Geldmacher DS, Herrup K (2001) DNA replication precedes neuronal cell death in Alzheimer’s disease. J Neurosci 21(8):2661–2668. 10.1523/JNEUROSCI.21-08-02661.2001 11306619 10.1523/JNEUROSCI.21-08-02661.2001 PMC6762514

[CR72] Yang LB, Lindholm K, Yan R, Citron M, Xia W, Yang XL, Beach T, Sue L, Wong P, Price D, Li R, Shen Y (2003) Elevated beta-secretase expression and enzymatic activity detected in sporadic Alzheimer disease. Nat Med 9(1):3–4. 10.1038/nm0103-3 12514700 10.1038/nm0103-3

[CR73] Yurov YB, Iourov IY, Vorsanova SG, Liehr T, Kolotii AD, Kutsev SI, Pellestor F, Beresheva AK, Demidova IA, Kravets VS, Monakhov VV, Soloviev IV (2007) Aneuploidy and confined chromosomal mosaicism in the developing human brain. PLoS ONE 2(6):e558. 10.1371/journal.pone.0000558 17593959 10.1371/journal.pone.0000558 PMC1891435

[CR74] Yurov YB, Vorsanova SG, Iourov IY (2009) GIN‘n’CIN hypothesis of brain aging: deciphering the role of somatic genetic instabilities and neural aneuploidy during ontogeny. Mol Cytogenet 2:23. 10.1186/1755-8166-2-23 19939257 10.1186/1755-8166-2-23 PMC2787505

[CR75] Yurov YB, Vorsanova SG, Iourov IY (2010) Ontogenetic variation of the human genome. Curr Genomics 11(6):420–425. 10.2174/138920210793175958 21358986 10.2174/138920210793175958 PMC3018722

[CR76] Yurov YB, Vorsanova SG, Liehr T, Kolotii AD, Iourov IY (2014) X chromosome aneuploidy in the Alzheimer’s disease brain. Mol Cytogenet 7(1):20. 10.1186/1755-8166-7-20 24602248 10.1186/1755-8166-7-20 PMC3995993

[CR77] Yurov YB, Vorsanova SG, Demidova IA, Kolotii AD, Soloviev IV, Iourov IY (2018) Mosaic brain aneuploidy in mental illnesses: an association of low-level post-zygotic aneuploidy with schizophrenia and comorbid psychiatric disorders. Curr Genomics 19(3):163–172. 10.2174/1389202918666170717154340 29606903 10.2174/1389202918666170717154340 PMC5850504

[CR78] Yurov YB, Vorsanova SG, Iourov IY (2019) Chromosome instability in the neurodegenerating brain. Front Genet 10:892. 10.3389/fgene.2019.00892 31616475 10.3389/fgene.2019.00892 PMC6764389

[CR79] Zhao C, Deng W, Gage FH (2008) Mechanisms and functional implications of adult neurogenesis. Cell 132(4):645–660. 10.1016/j.cell.2008.01.033 18295581 10.1016/j.cell.2008.01.033

[CR80] Zheng W, ZhuGe Q, Zhong M, Chen G, Shao B, Wang H, Mao X, Xie L, Jin K (2013) Neurogenesis in adult human brain after traumatic brain injury. J Neurotrauma 30(22):1872–1880. 10.1089/neu.2010.1579 21275797 10.1089/neu.2010.1579 PMC3815038

[CR81] Zhou L, Del Villar K, Dong Z, Miller CA (2004) Neurogenesis response to hypoxia-induced cell death: map kinase signal transduction mechanisms. Brain Res 1021(1):8–19. 10.1016/j.brainres.2004.05.115 15328027 10.1016/j.brainres.2004.05.115

