# Supplementary figures and images for: Aneuploidy is Linked to Neurological Phenotypes Through Oxidative Stress
Source: J Mol Neurosci. 2024 May 2;74(2):50. doi: 10.1007/s12031-024-02227-1 (PMC11062972; doi:10.1007/s12031-024-02227-1)

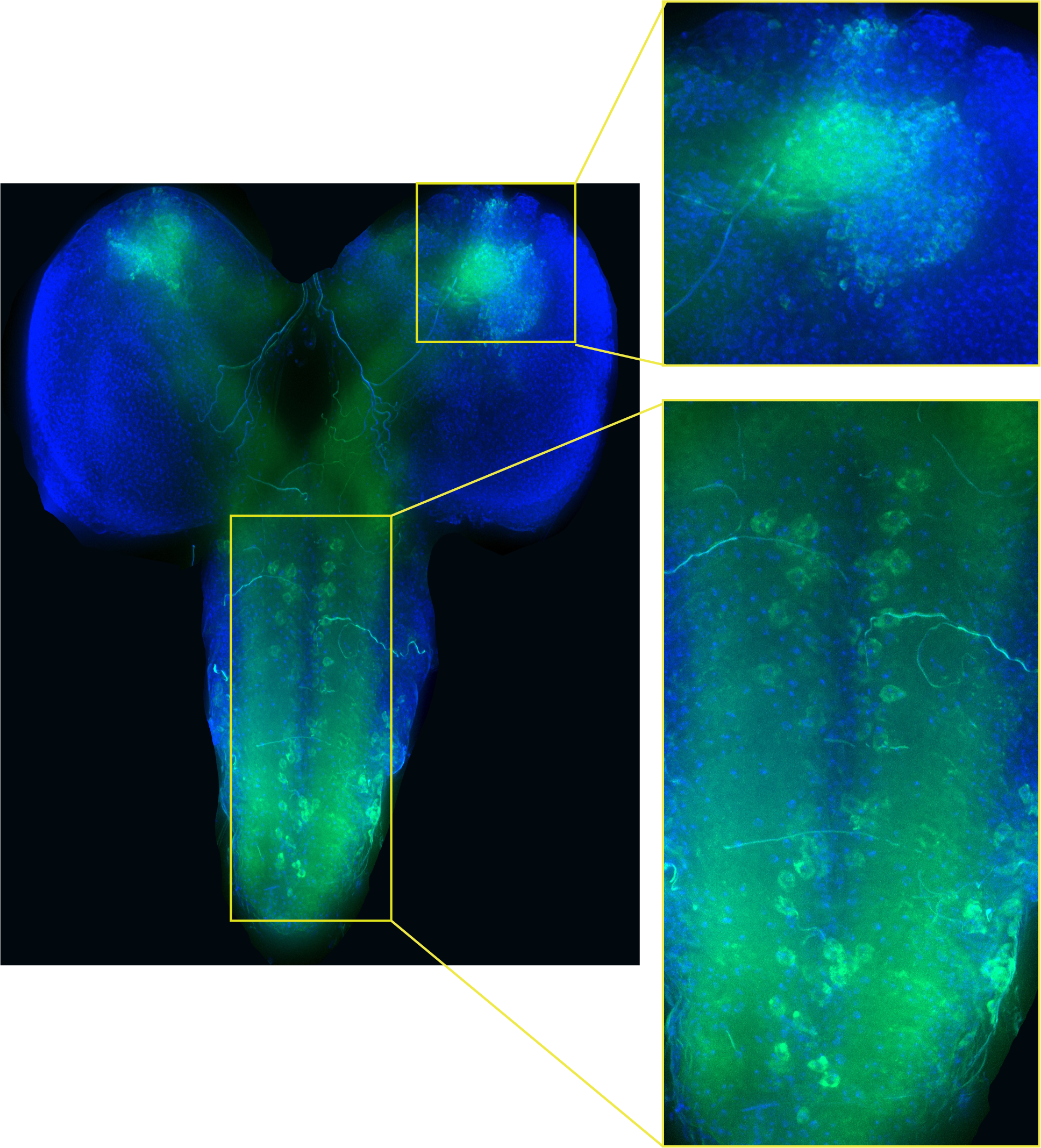

Supplement: Supplementary file 1 — Supplementary file1 Fig-1: The expression pattern of Gad1-Gal4 driver visualised by CD8-GFP in a 3rd instar Drosophila larval central nervous system.(TIF 12292 KB) [file 12031_2024_2227_MOESM1_ESM.tif]

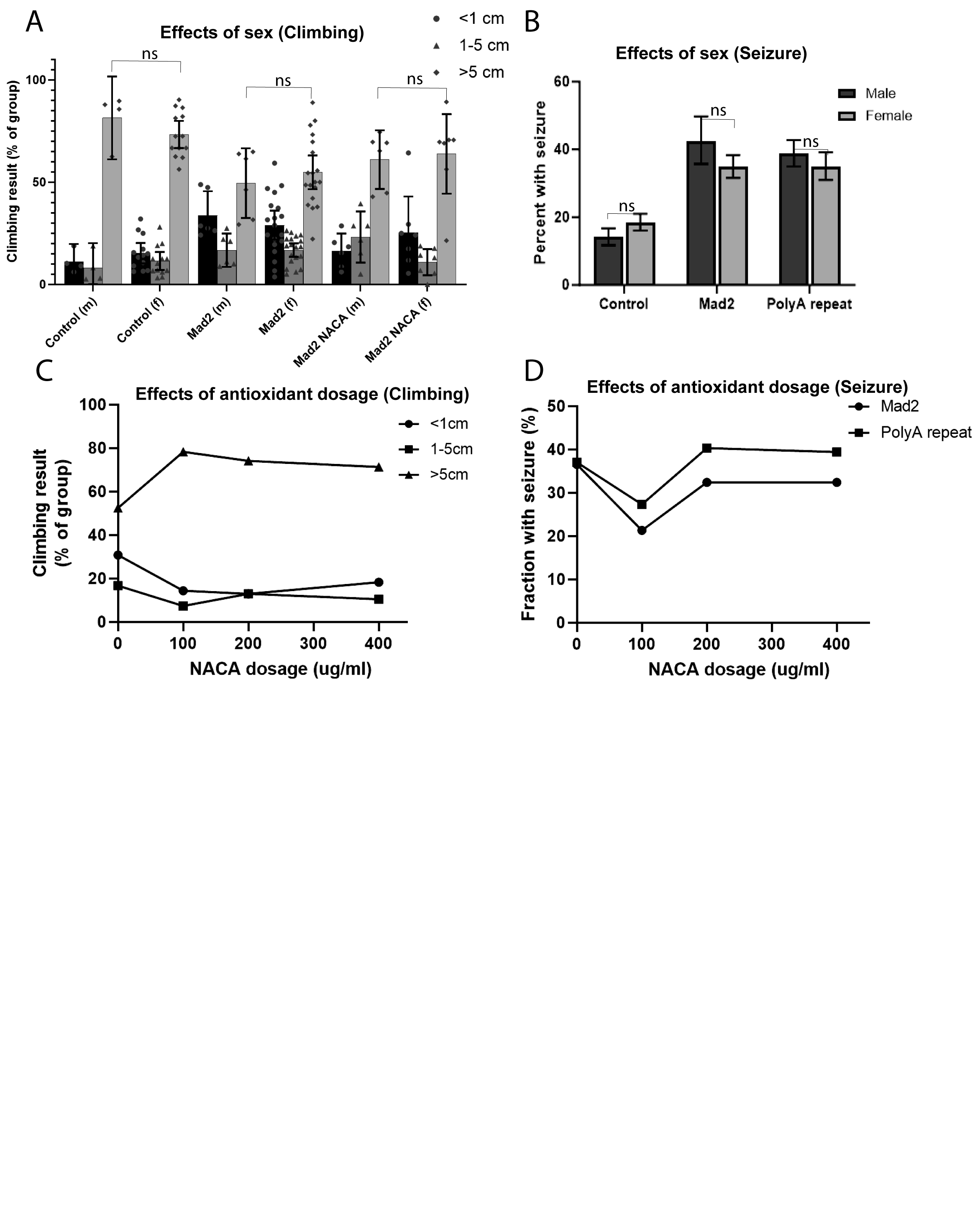

Supplement: Supplementary file 2 — Supplementary file2 Fig-2: The climbing performance and seizure-like phenotype in male vs females are shown in A (each group shown, avg >30 individuals per group) and B (n>270 for each genotype). No significant difference between male vs females were detected regarding the climbing performance and seizure like phenotypes. The effect of various doses of NACA feeding on Gad1-Gal4, UAS-Mad2-RNAi induced climbing defects and seizure like phenotypes are shown in C (n>230 for each dosage) and D (n>280 for each dosage). There was no significant improvement above 100 ug/ml for rescuing the climbing defects and seizure like-phenotype (C, D).(TIF 737 KB) [file 12031_2024_2227_MOESM2_ESM.tif]
